# Microscopy Cell Segmentation: Review and Benchmarking of Task-Specific and Foundation Models

**DOI:** 10.3390/jimaging12070297

**Published:** 2026-07-02

**Authors:** Diego Martí-Pérez, Valery Naranjo, Adrián Colomer

**Affiliations:** Instituto Universitario de Investigación en Tecnología Centrada en el Ser Humano (Human-Tech), Universitat Politècnica de València (UPV), Camino de Vera s/n, 46022 Valencia, Spain; vnaranjo@dcom.upv.es (V.N.); adcogra@upv.es (A.C.)

**Keywords:** deep learning, microscopy, cell segmentation, foundation models, machine learning, instance segmentation

## Abstract

Cell segmentation plays a key role in a wide range of biomedical imaging applications, from single-cell analysis to pathology assessment. While classical deep learning architectures such as U-Net, StarDist, and HoVer-Net have set strong baselines, their reliance on domain-specific training limits generalization across diverse microscopy modalities. The emergence of foundation models, particularly the Segment Anything Model (SAM) and its derivatives, has introduced a paradigm shift toward more universal and adaptable segmentation frameworks. In this review, we summarize key advances in microscopy cell segmentation, highlighting both traditional methods and recent foundation model-based approaches. Beyond surveying the literature, we present an experimental comparison of four representative models—our proposed YOLO-SAM, along with CellSAM, Cellpose-SAM, and StarDist—tested on both fluorescence and brightfield microscopy spanning diverse cell populations and shapes. Our findings illustrate trade-offs between accuracy, robustness, and adaptability, with foundation-based models showing particular promise for cross-domain performance. By combining a comprehensive review with systematic benchmarking, this work provides practical guidance for researchers and outlines current challenges and future opportunities in developing robust, generalizable cell segmentation methods for microscopy.

## 1. Introduction

Accurate cell segmentation is a foundational step in many biomedical image analysis pipelines, serving as the basis for quantifying cellular morphology, tracking dynamic behaviors, and understanding tissue architecture. It enables downstream tasks such as cell classification, phenotype analysis, and pathology assessment. In clinical research and diagnostics, precise delineation of individual cells from microscopy images is critical for detecting disease-related changes. For instance, in breast cancer, analyzing cell shapes, sizes, and spatial arrangements in histopathology or fluorescence images can reveal key features of tumor progression, heterogeneity, and response to treatment [1]. Manual annotation, while reliable, is labor-intensive and not scalable, especially with large datasets generated by high-throughput imaging platforms. This creates a strong demand for automated, robust segmentation methods that can support both research and clinical decision-making.

Microscopy imaging plays a central role in studying cellular morphology and behavior, with a variety of modalities offering different levels of contrast, resolution, and complexity. Fluorescence microscopy is widely used for its ability to highlight specific cellular components, but it often requires labeling, suffers from photobleaching, and may introduce imaging artifacts [2,3]. On the other hand, label-free techniques such as brightfield, phase-contrast, and Differential Interference Contrast (DIC) microscopy are more accessible and suitable for live-cell imaging, yet they produce low-contrast or artifact-prone images that make segmentation particularly challenging [4]. Variations in cell shape, density, and imaging conditions across and within these modalities further complicate segmentation tasks, requiring models to be robust, adaptable, and capable of generalizing across diverse visual features.

Cell segmentation tasks vary in complexity depending on the level of detail and dimensionality required. Semantic segmentation involves classifying each pixel as cell or background, without distinguishing between individual cells. In contrast, instance segmentation not only identifies cell pixels but also separates overlapping or adjacent cells into distinct objects, which is crucial for accurate quantification. Beyond 2D images, 3D segmentation extends analysis into volumetric data from confocal or light-sheet microscopy [5], introducing challenges related to anisotropic resolution and data size. Additionally, cell tracking across time-lapse sequences combines segmentation with temporal association, essential for studying cell migration, division, and dynamic processes [6]. Each of these tasks demands tailored algorithmic strategies and often requires domain-specific adaptations to handle noise, variability, and complex morphologies present in microscopy data.

Over the past decade, deep learning has transformed the field of biomedical image analysis, offering powerful tools for automating complex tasks like cell segmentation. Convolutional neural networks (CNNs), such as U-Net [7] and Mask R-CNN [8], have become standard for learning rich feature representations from annotated datasets. However, their performance requires task-specific training and sufficient labeled data. More recently, foundation models—large-scale, pretrained architectures like the Segment Anything Model (SAM) [9]—have emerged as versatile alternatives. These models are designed to generalize across domains with minimal task-specific tuning, opening new possibilities for applying Artificial Intelligence (AI) to diverse biomedical imaging problems. By leveraging massive datasets and powerful transformers, foundation models promise greater flexibility and robustness, even in challenging or low-data scenarios such as rare cell types or variable staining conditions.

Despite the rapid advancement of deep learning and the recent emergence of foundation models, there is still a lack of comprehensive evaluations comparing these models specifically in the context of cell segmentation in microscopy. Most existing surveys focus either on traditional segmentation methods or on general-purpose AI models without addressing their suitability for biological data. This review aims to bridge that gap by offering a focused comparison between classical and modern approaches, with a particular emphasis on foundation models like SAM and their adaptations for cell segmentation.

In addition to reviewing the current literature, we conducted a systematic experimental evaluation across multiple microscopy datasets containing brightfield and fluorescence images of different cell types. To provide a comprehensive assessment across the current methodological landscape, we benchmarked four representative models selected to span distinct architectural paradigms: StarDist [10] serves as the baseline for traditional, fully localized geometric modeling, while the emerging trend of foundation-model adaptation is represented by three approaches: CellSAM [11] (strong zero-shot performance), Cellpose-SAM [12] (hybrid architecture with strong generalization capabilities), and our proposed YOLO-SAM framework (decoupled local detection paired with a foundation decoder for segmentation). This evaluation aims to provide practical guidance for researchers and practitioners in selecting appropriate segmentation tools based on their specific annotation and computational bounds, while also highlighting the current limitations and opportunities that remain for future methodological advances.

## 2. Review

### 2.1. Trends in Publications and Research Focus

The task of cell segmentation in microscopy has become a key area of research in biomedical image analysis given its essential role in quantifying cellular phenotypes, tracking dynamics, and supporting diagnostic workflows. Over the past two decades, this field has gone through a significant evolution from the use of classical image processing methods to the adoption of modern deep learning techniques. This reflects, not only technological advancements in microscopy and computation, but also an increasing demand for scalable and accurate analysis in high-throughput biology and clinical pathology.

In the early 2000s, segmentation tasks relied heavily on traditional computer vision algorithms. These included global and adaptive thresholding techniques such as Otsu’s method [13], edge detection [14], watershed-based segmentation [15], and morphological operations (useful in tasks like noise removal or shape extraction). Such methods were often combined with manual feature extraction and simple machine learning classifiers, like support vector machines [16] and random forests [17]. While these approaches worked reasonably well on clean and high-contrast images, they struggled in cases with dense cellular clustering, diverse cell shapes, and noisy backgrounds. Such conditions are common in modalities like brightfield and phase-contrast microscopy [4]. During this phase, tools like CellProfiler [18] were instrumental as they provided a flexible, open-source platform that enabled experts to design image analysis workflows combining segmentation, feature extraction, and statistical quantification. It helped establish reproducible pipelines for large-scale biological experiments, such as RNAi screening [19] and early high-content phenotypic profiling works [20].

With the advances in microscopy and the growth of high-throughput imaging with techniques such as fluorescence labeling, live-cell imaging, and volumetric scanning, the complexity and quantity of image data increased substantially. This highlighted the limitations of previous segmentation approaches and provoked a shift towards data-driven methods. The introduction of deep learning into biomedical image analysis in the mid-2010s was a turning point. A major advancement was the U-Net architecture [7], which was designed specifically for biomedical segmentation. Its encoder–decoder structure with skip connections enabled precise localization while leveraging multi-scale features, and it showed strong performance with relatively few labeled samples. U-Net became the basis for many other variants and extensions across microscopy, histology, and other medical imaging domains [21,22].

Throughout the late 2010s and early 2020s, a variety of deep learning models were proposed to address the main challenges in microscopy. These include dealing with dense cellular clustering [23,24], segmenting overlapping or irregularly shaped cells [10], and adapting to multiple imaging modalities [25]. Deep learning was also extended to 3D segmentation [26] and cell tracking in time-lapse data [6]. A growing number of public datasets and community benchmarks supported these developments. Challenges like the Data Science Bowl 2018 [27], the ISBI Cell Tracking Challenge [28] or the NeurIPS 2022 Cell Segmentation Challenge [29] offered high-quality annotations and standardized evaluation metrics. These resources accelerated the research progress by enabling reproducible comparisons and catalyzing community innovation.

More recently, between 2023 and 2025, there has been a rise in methods using foundation models and multimodal learning to further improve segmentation accuracy and generalization across diverse datasets. The combination of vision transformers with convolutional architectures have demonstrated superior performance on complex tasks involving heterogeneous cell populations and varying imaging conditions [30]. In addition, self-supervised and few-shot learning approaches have gained attention, addressing the problem of limited annotated training data by enabling models to learn robust representations directly from unlabeled images [31,32]. These innovations have established a trend towards more flexible, scalable, and generalizable segmentation methodologies, pushing the boundaries of biological image analysis.

**Figure 1:**
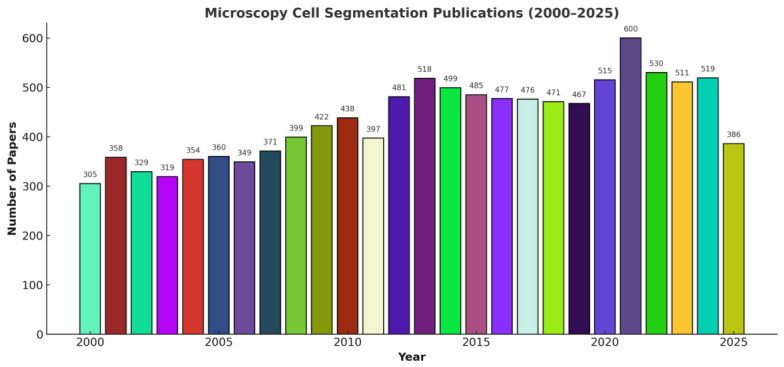
Bibliometric trend of publications related to microscopy cell segmentation (2000–2025). The y-axis represents the total number of publications identified via PubMed using the search query: (“microscopy” AND “cell segmentation”).

The growing attention to cell segmentation in microscopy is evident from the steady rise in scientific publications over the past two decades. A PubMed query using the terms “microscopy” AND “cell segmentation” returned 305 works in the year 2000 compared to peaks above 600 in 2021, with publication counts remaining above 500 in subsequent years (see Figure 1). This surge is particularly marked after 2015, coinciding with the widespread adoption of convolutional neural networks, transfer learning, and self-supervised learning in biomedical imaging [33,34]. The trend highlights a clear methodological shift from classical image processing approaches to deep learning-based strategies. More recently, research has begun to explore foundation models and multimodal frameworks, reflecting the field’s continuous push toward robust, generalizable, and biologically meaningful cell segmentation.

### 2.2. Applications of Cell Segmentation in Biomedical Research

Cell segmentation is a foundational component of biomedical image analysis. It enables multiple downstream applications in both research and clinical domains. Accurate delineation of individual cells and nuclei provides essential information about morphology, spatial organization, and temporal dynamics, which can be exploited in fields like cancer diagnostics, drug screening, and developmental biology.

In cancer, precise nuclear and cytoplasmic segmentation is utilized to extract features like size, shape, and texture that correlate with malignancy, mitotic activity, and tumor grade [35,36]. Concretely, histopathology workflows use instance segmentation for tumor boundary delineation, detection of tumor-infiltrating lymphocytes, and gland segmentation in prostate and breast cancer tissues. By automating these tasks, segmentation reduces inter-observer variability and significantly speeds up diagnostic processes.

In drug discovery and high-content screening, segmentation allows the quantification of phenotypic responses at a single-cell level. Tools such as CellProfiler [18] have made it possible to perform large-scale profiling of cellular morphology in response to drug perturbations, facilitating mode-of-action prediction and toxicity assessment [37].

Segmentation is also crucial in developmental and stem cell biology, where tracking of cells over time in 3D images allows lineage tracing and understanding of morphogenetic processes [28]. Similarly, in neuroscience, nuclear and soma segmentation is used to analyze cell distributions, layer structures, and pathological changes in brain tissue.

Recent progress in spatial omics technologies, such as spatial transcriptomics and multiplexed imaging, depend on cell segmentation to map molecular data to individual cells, enabling spatially resolved single-cell analysis [38]. This integration of imaging and genomics needs highly accurate segmentation, particularly in densely packed tissues with diverse cell types.

Overall, cell segmentation functions as the bridge between raw microscopy images and quantitative biological insight. Its applications are increasingly diverse and key for modern biomedical research.

### 2.3. Microscopy Techniques

Microscopy-based biomedical research encompasses a wide range of cell types, including cultured mammalian cells, stem cells, microbial organisms, and tissue biopsies. Each of them contains different morphological features and presents unique imaging challenges. For example, breast cancer cell lines such as MCF-7 and T47D are widely used in cancer biology to investigate tumor progression and drug responses [39,40]. Meanwhile, cells like Staphylococcus aureus serve as a key model in microbiology and infectious disease research [41]. Additionally, tissue sections introduce further complexity due to their dense and diverse cellular composition [1].

Addressing this wide variety of cellular features required the development of multiple microscopy approaches tailored to different needs. Early advances in microscopy began with transmitted light techniques such as phase-contrast microscopy [42], polarized light microscopy [43], and differential interference contrast (DIC) microscopy [44]. These label-free modalities enhanced the visibility of live cells by increasing intrinsic contrast without the need for staining. Although they represented a significant improvement for cell biology research, these techniques posed some limitations for automated image analysis.

Over time, microscopy techniques have developed into two primary categories: labeled and label-free imaging. Label-free methods, such as brightfield and phase-contrast microscopy, have been key for live-cell observation due to their simplicity and non-invasive nature. However, these modalities often suffer from low contrast and less clear cellular features, which present significant challenges for computational analysis and segmentation [45]. In contrast, the rise of labeled imaging, most notably fluorescence microscopy, revolutionized cell biology by enabling the visualization of specific biomolecular structures. This distinction between label-free and labeled approaches continues to shape modern imaging strategies, particularly in how image data is interpreted and processed by automated pipelines.

Fluorescence microscopy remains one of the most powerful and used tools in modern cell biology, offering specific and dynamic visualization of cellular structures and processes. This technique uses fluorescent probes like dyes, genetically encoded fluorescent proteins, and targeted antibodies. These bind selectively to biomolecules such as proteins, lipids, or ions [46]. This molecular specificity has enabled major advances in understanding cellular organization, protein localization, and real-time signaling events.

To overcome the diffraction limit of conventional fluorescence microscopy, several super-resolution techniques have been developed. Methods such as Photoactivated Localization Microscopy (PALM) [3], Stimulated Emission Depletion (STED) [2], and Stochastic Optical Reconstruction Microscopy (STORM) [47] allow imaging at the nanometer scale, making it possible to study subcellular architectures with higher detail.

Building on these advances, fluorescence microscopy has further evolved to address limitations in imaging depth, speed, and live-cell compatibility. Light-sheet fluorescence microscopy (LSFM) [5] and, in particular, lattice light-sheet microscopy (LLSM) [48] allow fast and volumetric imaging of live cells and tissues with minimal photodamage. These techniques have enabled the capture of dynamic biological processes in three dimensions over time, with recent applications such as embryogenesis [49], neural activity mapping [50], and immune cell dynamics [51].

More recently, fluorescence microscopy has advanced through high-content and multiplexed imaging strategies. Techniques such as spectral imaging, molecular barcoding, and sequential fluorescence in situ hybridization (seqFISH) [52,53] allow for the simultaneous detection of many molecular species within the same sample. In addition, the integration of deep learning and artificial intelligence is rapidly transforming fluorescence microscopy data analysis. AI-driven methods enhance image denoising, resolution, and segmentation [54,55]. This facilitates the reconstruction of high-quality images from low-exposure data, reduction of phototoxicity, and even prediction of fluorescence labels from transmitted-light images. Together, these advances demonstrate how fluorescence microscopy keeps pushing the boundaries of cellular imaging.

### 2.4. Computer Vision Tasks

Artificial intelligence has revolutionized the analysis of microscopy data by enabling a broad spectrum of tasks, from low-level image interpretation to complex biological insight. The main Computer Vision (CV) tasks in this domain include object detection, classification, semantic and instance segmentation, anomaly detection, cell tracking, and 3D segmentation and reconstruction. Each of these plays an important role in biomedical image analysis, enabling processes like quality control, phenotyping, and the modeling of dynamic biological processes.

Object Detection consists of identifying and localizing individual cells or structures using bounding boxes. This tasks often serves as a first step for more complex tasks such as segmentation or tracking. While traditional detection relied on handcrafted features and region proposal methods [56], deep learning-based detectors such as the Single Shot Multibox Detector (SSD) [57] and You Only Look Once (YOLO) [58] revolutionized the field by achieving real-time and end-to-end detection in a single network pass. Even though these models were originally developed for natural scenes, they have been adapted for microscopy and histopathology images to detect nuclei, mitotic events, and tissue abnormalities [59]. Object detection plays a key role in applications like mitosis detection in cancer diagnostics and identifying regions of interest for downstream analysis [33].

Image Classification is one of the most fundamental tasks, where models are trained to assign discrete labels to full images or certain regions of interest. Common applications in biomedical contexts involve classifying between cancerous and non-cancerous tissue samples, identifying different cell types, detecting stages of infection, or predicting cellular responses to treatments. In the past, most of the methodologies contained image descriptors and classical machine learning methods, such as random forests or support vector machines [60]. However, they have been replaced by convolutional neural networks (CNNs) given their higher ability to learn feature representations. In high-content screening workflows, classification models are often used for automated phenotypic profiling, supporting large-scale drug discovery and toxicity studies [61].

More recent trends in microscopy classification include the adoption of transformer-based architectures [62], self-supervised learning [63], and multimodal fusion (e.g., combining image data with metadata or gene expression) [64]. These approaches aim to enhance generalization across datasets and experimental conditions, which is the main challenge in the field due to batch effects and biological variability.

Additionally, explainability is a growing research focus. Saliency maps, class activation maps (CAMs), and other visualization techniques are used to highlight those regions that contribute most to the model’s decision, supporting interpretability in clinical or biological contexts [65,66].

Semantic Segmentation provides pixel-level classification of microscopy images, assigning each pixel to a specific class, such as nucleus, cytoplasm, background, or tissue type. This task is particularly important for morphometric analyses, allowing researchers to quantify features like cell size, shape, and spatial organization. A major advancement in this area was the development of U-Net [7], which introduced a symmetric encoder–decoder architecture with skip connections, enabling accurate localization and robust generalization from relatively small datasets. Since then, U-Net has become the baseline in the field, inspiring numerous adaptations and extensions incorporating deeper backbones, residual connections, attention modules, and adversarial refinement strategies [67,68]. Complementary architectures such as DeepLab [69] have also demonstrated strong performance, particularly with their use of dilated convolutions and fully connected conditional random fields (CRFs) for improving object boundary precision. These models have been adapted for biomedical images, where capturing fine details such as cell borders is essential.

More recently, transformer-based architectures and transfer learning have shown promising results for generalization across datasets. Models like UNETR [70] and SegFormer [71] leverage self-attention mechanisms, improving segmentation accuracy in complex biomedical samples. These architectures, combined with transfer learning strategies, have shown strong performance even with limited annotated data. For instance, comparative studies of deep transfer learning models have demonstrated their potential for generalization and domain adaptation [72]. Such approaches reduce the dependence on large annotated datasets while enhancing cross-domain robustness.

Semantic segmentation has now been applied to a wide range of imaging modalities, from fluorescence microscopy of cultured cells to brightfield and histological tissue sections. Ongoing challenges, like staining variability, imaging artifacts, and domain shifts across labs and instruments, have grown interest in unsupervised domain adaptation and self-supervised pretraining techniques.

**Figure 2:**
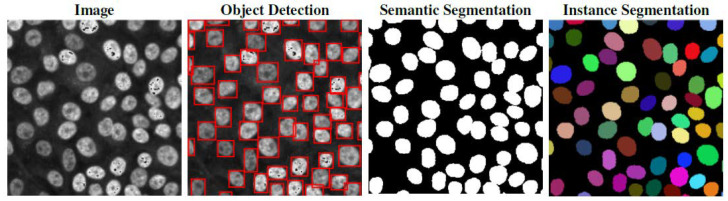
Illustration of different computer vision tasks in biomedical imaging.

Instance segmentation goes beyond semantic segmentation by not only classifying each pixel but also distinguishing individual objects within the same class (see Figure 2). This detail is critical for analyzing densely packed or overlapping cells. In single-cell biology, instance segmentation enables accurate quantification of cell counts, spatial organization, and cellular heterogeneity.

State-of-the-art approaches in cell instance segmentation have been built upon general frameworks such as Mask R-CNN [8], adapted to biological imaging contexts. Domain-specific tools like Cellpose [23] and StarDist [10] incorporated tailored strategies to accurately delineate cell boundaries even under challenging imaging conditions. The segmented instances produced by these models often provide the starting point for subsequent analyses, such as cell tracking, lineage reconstruction, and phenotypic profiling. A detailed discussion of instance segmentation methods and their applications is provided in the next section.

Anomaly Detection aims to identify rare, unexpected, or abnormal patterns within microscopy images. Such anomalies may correspond to unusual phenotypes, mitotic defects, apoptotic bodies, or imaging artifacts. Due to their rarity in most datasets, anomaly detection often relies on unsupervised or self-supervised learning techniques. Methods such as autoencoders [73], generative adversarial networks (GANs) [74], and contrastive learning [75] are frequently employed to model normal data distributions, enabling us to flag deviations as anomalies. In pathology, anomaly detection has been applied to identify tumor regions in large histological images [76] and detect poorly differentiated cells in hematological samples [77].

Cell Tracking over time is essential for studying dynamic biological processes such as migration, proliferation, differentiation, and apoptosis. This task involves identifying and associating cells across consecutive time-lapse frames to reconstruct their temporal trajectories. Traditional approaches have relied on object detection and motion prediction algorithms, such as Kalman filters or nearest-neighbor heuristics [78,79]. However, deep learning-based tracking methods, including recurrent neural networks (RNNs) [80] and graph neural networks [81], have shown substantial improvements under conditions such as cell division, merging, or sudden changes in shape. For example, deep learning models have been applied to track breast cancer cells in migration assays, even under challenging conditions with occlusions and rapid motion [6].

More recently, models like DeepSea [82], Cellpose [23], and Omnipose [83] have integrated segmentation and tracking capabilities into unified pipelines. These models offer robustness across diverse imaging modalities and cell types. In particular, Omnipose extends Cellpose by improving segmentation of irregularly shape cells, making it especially valuable for bacterial and morphologically diverse datasets.

Three-dimensional Segmentation and Volumetric Analysis refers to the analysis of three-dimensional imaging data acquired from modalities such as confocal microscopy, light-sheet fluorescence microscopy, and electron microscopy. These techniques produce volumetric datasets where biological structures extend across multiple optical sections. Accurate 3D segmentation and reconstruction are essential for quantitative analysis of tissue architecture, subcellular organization, and organoid morphology [84]. To address this, 2D deep learning models have been extended into 3D, with architectures like 3D U-Net [26] and V-Net [85]. These approaches employ volumetric convolutions to capture spatial context in all three dimensions. Due to the high computational demands of volumetric data, specialized strategies such as patch-based training, tiling, multi-scale approaches, and hybrid 2D/3D pipelines are often adopted [22,86]. These models have provided detailed insights of complex biological system. For instance, they have been applied to tasks like segmentation of cell nuclei in z-stacks [87], synapse detection in electron microscopy volumes [88], and neuronal circuit tracing [89].

### 2.5. Instance Segmentation

As introduced in the previous section, instance segmentation refers to the task of identifying and delineating individual objects within an image, assigning unique labels to each detected instance. Table 1 provides an overview of the most representative models in this area.

Mask R-CNN [8] sets a strong foundation by combining object detection and pixel-level segmentation using a region-based approach. Since then, numerous architectures have emerged, focusing on refining mask quality, improving instance separation, or enhancing efficiency. Notably, earlier works such as adversarial and recurrent models [90] employed convolutional LSTM structures to capture spatial and temporal features, marking some of the first deep learning attempts in biomedical instance segmentation.

In histopathology, instance segmentation is vital for identifying nuclei, glands, and tissue compartments. These are key for cancer grading, tumor analysis, and digital pathology pipelines. HoVer-Net [91] emerged as a landmark model in this field by predicting horizontal and vertical distance maps to better separate clustered nuclei, achieving strong performance across several histological datasets. Meanwhile, Mesmer [24], trained in multiplexed images, demonstrated strong generalizability across tissues and imaging protocols, enabling the automated extraction of key cellular characteristics, such as subcellular location of protein signal.

In microscopy, where images include diverse cell types acquired via various modalities like fluorescence, brightfield, or phase-contrast, the instance segmentation task presents unique challenges. Some of them are overlapping cells, low contrast, and highly variable shapes. Custom variants of Mask R-CNN and U-Net hybrids have been widely used to balance precise localization with segmentation accuracy. More specialized models like StarDist [10] introduced a star-convex polygon representation for segmenting nuclei in fluorescence images, greatly improving accuracy in crowded environments. Cellpose [23] leveraged vector flow fields to robustly delineate cells across multiple modalities and morphologies, later extended by Cellpose 2.0 [92] and Cellpose 3.0 [97], which offer interactive training and support for 3D segmentation. BriFiSeg [94] addressed the complexity of gland segmentation through a multi-scale approach tailored to accommodate variable gland morphologies. CPP-Net [95] proposed a contour proposal network to enhance instance segmentation in densely packed cell images. LACSS [93] introduced a weakly supervised framework leveraging image-level annotations for effective segmentation. Omnipose [83] enhanced performance on bacterial and irregularly shaped cells by modeling more flexible object contours and improving boundary localization. Cellulus [96] recently combined self-supervised learning and multi-scale features for improved segmentation of heterogeneous microscopy datasets.

As segmentation demands continue to grow, particularly in applications with limited annotations or new imaging modalities, recent research has turned toward foundation models. These large-scale pretrained models aim to provide general-purpose segmentation capabilities with minimal fine-tuning. In the following section, we explore the emergence of foundation models in biomedical image segmentation and their adaptation to microscopy data.

### 2.6. Foundation Models

Foundation models represent a transformative paradigm in computer vision, defined by their large scale, versatility, and strong generalization capabilities across diverse tasks with minimal fine-tuning. These models are typically pre-trained on massive datasets using self-supervised learning objectives, enabling them to learn broad, general-purpose visual representations that can be adapted to downstream applications such as segmentation, classification, and detection through prompt-based or lightweight tuning strategies [98]. Many of these models leverage transformer-based architectures, including the Vision Transformer (ViT), which has shown remarkable ability to capture long-range dependencies and contextual information in images, further boosting model generalization [99]. This architectural shift plays a key role in enabling foundation models to transfer effectively across domains, even when domain-specific annotated data is scarce.

A notable example is the Segment Anything Model (SAM), developed by Meta AI [9]. Trained on over one billion masks from 11 million images, SAM introduces a highly flexible prompting interface that accepts inputs such as points, bounding boxes, or masks. This design supports both interactive and automated segmentation across an enormous wide range of image types and domains. SAM’s impressive zero-shot generalization capabilities make it particularly appealing for biomedical applications, where labeled data is often limited and manual annotation is very time-consuming.

Building on SAM’s foundation, researchers have begun tailoring it to better address the unique challenges of biomedical images (see Table 2). For instance, Cellpose-SAM combines SAM’s mask generation strengths with the domain-specific expertise of Cellpose [12]. Similarly, Cell-SAM employs domain-specific training strategies to refine segmentation outputs, accommodating the dense, low-contrast, and morphologically diverse structures characteristic of cellular microscopy [11]. MicroSAM focuses specially on micro-scale cellular details, enhancing sensitivity to detect contours and faint boundaries frequently found in brightfield and label-free imaging modalities [100]. Vista 2D [101] is another recent adaptation that improves segmentation of 2D microscopy images by integrating SAM with contrastive learning techniques, further boosting robustness under challenging imaging conditions. These adaptations underscore that while SAM provides a powerful generalist base, incorporating biological priors and specialized knowledge is crucial for achieving high-precision biomedical segmentation.

Despite these advances, challenges remain, including improving model robustness to noisy or low-quality images and reducing false positives in densely packed cellular environments. Nevertheless, foundation models are rapidly becoming indispensable tools in biological image analysis. As ongoing research continues to incorporate domain-specific refinements, these models are expected to surpass traditional segmentation methods in accuracy, adaptability, and efficiency.

### 2.7. Datasets

High-quality datasets are fundamental to the development and benchmarking of instance segmentation algorithms in biomedical research. To support effective model training, such datasets must include high-resolution images alongside instance-level annotations, where each individual cell (or nucleus) is assigned a unique and non-overlapping mask. These annotations are crucial for evaluating, not just whether the correct regions are segmented, but also whether individual cells are properly separated, especially in crowded or complex tissue environments.

However, creating reliable instance segmentation datasets in microscopy presents multiple challenges. For instance, manual annotation of individual cells requires substantial domain expertise to accurately delineate cell boundaries, particularly in cases involving overlapping structures, low contrast, or irregular shapes. This is further intensified in certain modalities like brightfield or phase-contrast microscopy, where boundaries are often poorly defined. In addition, annotation consistency across large datasets can be difficult to maintain due to inter-annotator variability and subjective interpretation of ambiguous boundaries. Such inconsistencies and label noise can reduce model generalizability, especially in those that rely heavily on clean supervision.

Early progress in dataset development was stronger in histology than microscopy, particularly for tasks involving nuclear and tissue segmentation. In 2016, a breast cancer histopathology dataset that remains influential in studies involving H&E-stained tissue classification and segmentation [102]. A few years later, the MoNuSeg dataset [103] extended this effort by providing manually annotated nuclear masks across diverse tissue types, serving as a benchmark for both segmentation and generalization studies. More recently, MoNuSAC [104] and Pannuke [105] datasets introduced multi-class instance-level annotations of nuclei, enabling evaluation of instance segmentation performance across multiple nuclear categories. Similarly, NuCLS delivered large-scale annotations of nuclei in breast cancer slides, combining crowd-sourced and expert-labeled data to improve label quality and scale [106]. Collectively, these datasets have helped establish benchmarks for deep learning models applied to clinical and histopathological data.

Microscopy cell segmentation research has been propelled by the release of numerous publicly available datasets encompassing a wide range of imaging modalities, cell types, and annotation styles. Table 3 summarizes representative microscopy datasets for segmentation, highlighting the imaging modalities. One of the earlier widely adopted resources was the Data Science Bowl (DSB18) dataset [27], introduced through a Kaggle challenge and offering annotated fluorescence microscopy images of nuclei from diverse experimental settings. It remains a foundational benchmark for nuclear segmentation. The Cellpose dataset [23] expanded the diversity of available data by including a broad set of cell types imaged using fluorescence, brightfield, and phase-contrast microscopy. The dataset features hand-annotated masks curated for generalist model development across modalities. Around the same time, LIVECell [107] was introduced, providing high-resolution phase-contrast images across multiple live cell lines along with dense instance masks, designed to support segmentation and tracking in time-lapse imaging. In addition, TissueNet [24] extended these efforts to tissue-scale fluorescence microscopy, comprising tens of thousands of immunofluorescence images from human tissues spanning over 60 anatomical and disease contexts, and providing high-quality nuclear and whole-cell annotations to train robust and generalizable segmentation models.

Subsequently, the NeurIPS Cell Segmentation Challenge dataset [29] was created to evaluate segmentation models under cross-domain conditions. It includes images from various microscopy modalities, tissue types, and staining protocols, serving as a rigorous benchmark for domain generalization in instance segmentation tasks. In parallel, domain-specific datasets continued to emerge. DeepBacs [108] further advanced bacterial segmentation by including diverse species and imaging conditions—synthetic, brightfield, and phase-contrast—and offering finely detailed instance masks. It also introduced a suite of test sets designed to evaluate generalization across biological and technical domains. Complementing these, the EVICAN dataset [109] provides extensive brightfield images of mammalian cells, addressing challenges related to label-free segmentation with diverse morphologies and imaging conditions.

Together, these datasets form a comprehensive ecosystem for benchmarking instance segmentation in microscopy, ranging from traditional fluorescence and live-cell imaging to more challenging bacterial and tissue-level tasks. Their continued development supports the advancement of robust, generalizable segmentation algorithms for biomedical research.

Creating high-quality annotated datasets for cell segmentation remains a major challenge due to the need for expert labeling, especially with overlapping cells, heterogeneous tissues, and low-contrast modalities like brightfield or phase-contrast. Variability in annotations and label noise can hinder model performance and generalization. Limited availability of 3D and time-lapse annotated data further restricts progress. Competitions such as the Data Science Bowl [27], ISBI Cell Tracking Challenge [6], and NeurIPS Cell Segmentation Challenge [29] have helped by providing standardized datasets and benchmarks, yet the gap between existing datasets and the complexity of real-world microscopy remains significant.

### 2.8. Segmentation Evaluation Metrics

Evaluating the performance of instance segmentation models is essential to ensure reliable and reproducible results, especially in biomedical applications where accuracy in cell boundary detection directly impacts downstream analyses. Several standard metrics are commonly used to assess segmentation quality, each capturing different aspects of instance-level accuracy.

Intersection over Union (IoU) is a fundamental metric that quantifies the overlap between a predicted mask and the corresponding ground truth mask. It is defined as:

(1) $$\mathrm{IoU}=\frac{\left|A\cap B\right|}{\left|A\cup B\right|}$$

where *A* is the predicted object region and *B* is the ground truth region. A higher IoU indicates a better match between predicted and true cell boundaries. IoU is often used with a threshold to determine whether a predicted object is considered a true positive.

Dice Score is another widely used metric that measures the similarity between the predicted and ground truth masks. It is defined as:

(2) $$\mathrm{Dice}=\frac{2|A\cap B|}{\left|A|+|B\right|}$$

Like IoU, Dice score ranges from 0 to 1, where 1 indicates perfect overlap. Dice score is especially useful in biomedical segmentation tasks due to its sensitivity to both false positives and false negatives, making it suitable for imbalanced datasets.

F1-score at a specific IoU threshold (e.g., 0.5) is commonly used to balance precision and recall:

(3) $$F1=\frac{2\cdot \mathrm{Precision}\cdot \mathrm{Recall}}{\mathrm{Precision}+\mathrm{Recall}}$$

This metric is sensitive to both over-segmentation and under-segmentation, making it suitable for evaluating dense cellular environments.

Average Precision (AP) summarizes the precision–recall trade-off across different IoU thresholds. In instance segmentation, AP is often computed as the mean precision over a range of IoU thresholds, commonly from 0.5 to 0.95 in steps of 0.05, known as AP@[.5:.95]:

(4) $$\mathrm{AP}=\frac{1}{\left|T\right|}\sum_{\tau \in T}\int_{0}^{1}p\left(r;\tau \right)\;dr$$

where *r* denotes recall, p(r;τ) is the precision at a given recall under a specific threshold, and T={0.50,0.55,…,0.95} represents the set of evaluated Intersection-over-Union (IoU) thresholds. AP provides a comprehensive view of model performance by integrating both detection quality and segmentation accuracy. It is widely used in computer vision challenges such as COCO and adapted in biomedical evaluations.

Panoptic Quality (PQ) is a comprehensive metric that jointly evaluates segmentation quality and recognition performance. It combines the effects of true positives (TPs), false positives (FPs), and false negatives (FNs). PQ is calculated as:

(5) $$\mathrm{PQ}=\frac{\sum_{(p,g)\in TP}\mathrm{IoU}\left(p,g\right)}{\left|\mathrm{TP}\right|+\frac{1}{2}\left|\mathrm{FP}\right|+\frac{1}{2}\left|\mathrm{FN}\right|}$$

where *p* and *g* represent matched predicted and ground truth instances. PQ effectively balances object detection and segmentation quality, making it robust across complex datasets.

Aggregated Jaccard Index (AJI) is another widely used metric in biomedical image segmentation that accounts for the intersection and union of matched objects while penalizing unmatched false positives. It is defined as:

(6) $$\mathrm{AJI}=\frac{\sum_{i=1}^{K}\left|G_{i}\cap P_{\sigma (i)}\right|}{\sum_{i=1}^{K}|G_{i}\cup P_{\sigma (i)}|+\sum_{l\in U}\left|P_{l}\right|}$$

where $G_{i}$ is the *i*-th ground truth instance, $P_{\sigma (i)}$ is the corresponding predicted match, *U* is the set of unmatched predictions, and $P_{l}$ is an unmatched predicted object. AJI provides a global view of segmentation performance over the entire image and is especially useful for datasets with many touching or overlapping cells.

These metrics are typically computed over datasets such as DSB18 or the NeurIPS Cell Segmentation Challenge to allow fair comparisons across models. It is crucial to interpret these scores in the context of the biological task at hand, as small segmentation errors might have negligible or significant impact depending on the downstream application.

### 2.9. Challenges and Future Directions

Despite major advances in cell segmentation driven by deep learning, there are still numerous challenges that limit widespread deployment and generalization of existing methods. These challenges span data availability, domain adaptation, model scalability, evaluation consistency, and practical applicability in real-world biological workflows.

Data diversity and annotation efforts remain as main obstacles. While datasets like DSB18, the Cellpose dataset, and the NeurIPS Cell Segmentation Challenge have supported the development and benchmarking of new models, they often cover a narrow range of imaging modalities, staining protocols, and biological conditions. Manual annotation of instance segmentation masks is time-consuming and requires expert knowledge. These difficulties are compounded in 3D and time-lapse data, which are essential for understanding dynamic cellular processes but are still very underrepresented in public datasets.

Generalization and domain shift pose further difficulties. Many models perform well within the distribution of their training data but degrade significantly when applied to different cell types, imaging systems, or sample preparations. Although some generalist approaches such as Cellpose aim to address this issue, even these models often require fine-tuning or manual correction in unfamiliar contexts. Building segmentation tools that are robust across biological domains remains a central goal.

Scalability to complex data is also a limitation. While many models work well on 2D fluorescence images, they often struggle with 3D volumes, temporal sequences, or densely packed tissues. Segmentation in these settings requires both increased computational resources and specialized model architectures. Recent efforts have made progress toward integrating 3D instance segmentation and tracking, but unified models that work reliably across spatial and temporal scales are still lacking.

Evaluation inconsistencies are another source of difficulty in comparing models fairly. Different datasets often employ different annotation styles and use varied metrics such as Intersection-over-Union (IoU), average precision (AP), or F1-score, which makes direct comparisons challenging. Moreover, segmentation accuracy is not always indicative of biological utility: small errors in delineating boundaries can significantly affect downstream tasks like cell counting or spatial analysis in tissues.

To address these issues, the task of cell segmentation in microscopy images is moving toward several promising directions:

- Foundation models adapted to microscopy, such as microSAM or Cell-SAM, are emerging as a flexible solution to limited annotated data and domain-specific variation.
- Self-supervised learning and synthetic data generation are being explored to reduce annotation dependence and improve robustness.
- Uncertainty quantification and interpretability are gaining traction to support use in clinical and high-stakes biological research.
- End-to-end frameworks that link segmentation to downstream tasks (e.g., cell tracking, spatial analysis, classification) offer potential for more integrated analysis pipelines.

Instance segmentation plays a pivotal role in medical imaging, as it enables the precise delineation of individual cells, nuclei, or anatomical structures—an essential step for quantitative analysis, disease diagnosis, and treatment planning. Given the fragmented landscape of models, datasets, and evaluation practices in cell segmentation, a systematic comparison of representative state-of-the-art methods under controlled conditions is urgently needed. Such a benchmark would provide insights into how models perform across cell types and imaging modalities when trained and tested consistently. In the next section, we address this gap by comparing leading segmentation models—including traditional deep learning methods and recent hybrid approaches—on a curated set of microscopy datasets. Our aim is to establish a fair and transparent evaluation that can inform future model development and help practitioners choose appropriate tools for their specific biological tasks.

## 3. Experiments

### 3.1. Data

**Figure 3:**
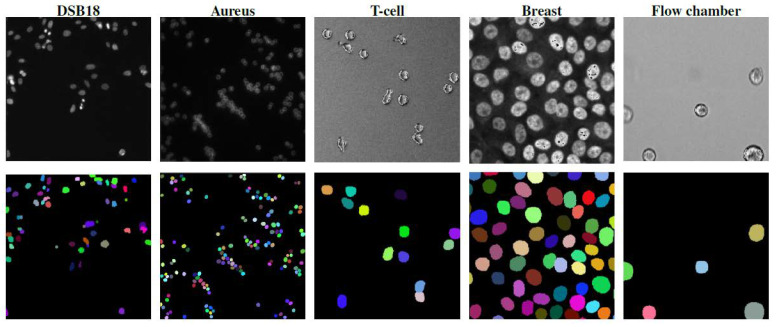
Representative samples from the datasets used in this study, with corresponding segmentation masks below each raw image.

To train our models, we combined three microscopy datasets spanning both brightfield and fluorescence modalities: the Data Science Bowl 2018 (DSB18) [27], Aureus [110], and T-cell [111] datasets. To evaluate generalization, we included two additional datasets not used in training: the Breast cancer [112] and Flow chamber [113] datasets. Together, this setup ensures assessment across familiar and cross-domain scenarios. Table 4 summarizes the datasets, modalities, and number of images used in both training and testing. Every dataset provided manually curated ground-truth masks, enabling reliable supervision during training and accurate evaluation. Representative examples of the raw images and their segmentation masks are shown in Figure 3.

- DSB18 [27]: A large collection of segmented nuclei acquired under diverse conditions, varying in cell type, magnification, and imaging modality (brightfield and fluorescence). It is specifically designed to test algorithmic generalization across heterogeneous data and is widely used as a benchmark in biomedical segmentation.
- Aureus [110]: Comprises paired DIC and Nile Red fluorescence images of *Staphylococcus aureus*, capturing bacterial morphology across dual modalities. The presence of densely packed cells makes accurate segmentation particularly challenging in this dataset.
- T-cell [111]: Contains brightfield microscopy images of migrating T-cells with masks obtained through manual segmentation (https://github.com/HenriquesLab/ZeroCostDL4Mic/wiki/Stardist (accessed on 19 May 2025). The original images (1024 × 1024) were cropped to 256 × 256 for consistency.
- Breast cancer [112]: Fluorescence microscopy images (SiR-DNA), cropped to 256 × 256 for consistency. Included to evaluate model generalization in the fluorescence domain.
- Flow chamber [113]: Brightfield time-lapse sequences of cells under flow conditions. This dataset allows testing model robustness in dynamic environments and generalization to unseen brightfield data.

This setup allows us to assess performance not only on familiar modalities but also in cross-domain scenarios, simulating real-world variability in microscopy data. Such diversity ensures that our evaluation goes beyond dataset-specific optimization, providing a more realistic benchmark of each model’s robustness and potential for broader biomedical applications.

### 3.2. Preprocessing and Software

All datasets were preprocessed with a standardized pipeline to ensure consistency across imaging modalities and cell types. Images were center-cropped to 256 × 256 pixels to match the input requirements of the models and to provide uniform spatial context. Intensity normalization was applied to reduce variability arising from different acquisition conditions, thereby facilitating fair comparisons across datasets.

No data augmentation (e.g., flips, rotations, or intensity perturbations) was applied during training or testing, as the primary goal was to evaluate model generalization on unaltered microscopy data. While this isolates raw cross-domain adaptability, it introduces an asymmetric impact. Specialized models like StarDist rely heavily on augmentation to learn orientation and intensity invariances, meaning this omission may suppress their performance. Conversely, foundation frameworks like YOLO-SAM inherit spatial robustness from massive pre-training corpora, making them less reliant on target-side data manipulation. Images were formatted according to the model specifications: converted to grayscale when required or retained as multi-channel inputs when applicable. Ground-truth annotations were uniformly represented as instance-level binary masks, ensuring compatibility between datasets and enabling consistent evaluation across all models.

All experiments were implemented in Python 3.12.3 using PyTorch 2.4.1+cu121 as the primary deep learning framework. Training and inference were conducted on an NVIDIA A100-SXM4 GPU with 40 GB memory.

### 3.3. Models

We evaluate four representative segmentation models that span classical, specialized, and foundation-model approaches. These include: (i) StarDist, a geometry-inspired deep learning model for star-convex objects; (ii) CellSAM, a foundation model adapted to microscopy data; (iii) Cellpose-SAM, a hybrid method combining the generalist strengths of SAM with the robustness of Cellpose; (iv) YOLO-SAM, our proposed method that integrates YOLO-based prompts with SAM for enhanced instance segmentation. For clarity, we separate our proposed method (YOLO-SAM) from the compared baselines.

#### 3.3.1. Proposed Method

**Figure 4:**
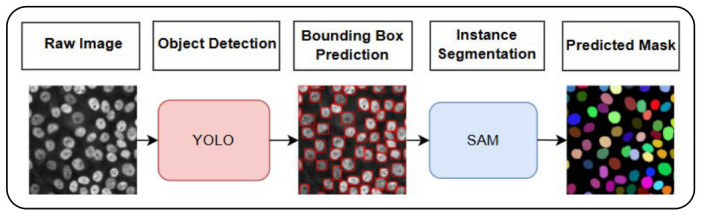
Workflow of the proposed YOLO-SAM framework for automated cell segmentation. First, the YOLO detector identifies cell regions and generates bounding box prompts, which are then passed together with the raw image to the Segment Anything Model (SAM) for precise instance mask prediction.

Our approach integrates YOLO-based object detection with SAM’s segmentation capabilities to achieve fully automated and high-precision cell segmentation (see Figure 4). Rather than relying on manually provided or heuristic prompts, bounding box prompts are generated automatically by a trained YOLO detector and then passed to SAM for mask prediction. This integration bridges two complementary paradigms—detection and segmentation—resulting in improved instance separation and accuracy on microscopy datasets.

While coupling object detection with foundation-scale segmentation aligns conceptually with traditional “Detect-to-Segment” workflows, adapting this pipeline to micro-scale cellular data requires resolving severe instance cross-talk. In dense cell populations, standard macroscopic bounding boxes naturally encapsulate fragments of adjacent cells, causing SAM’s default mask decoder to bleed across distinct boundaries. To mitigate this without modifying SAM’s core architecture, our implementation leverages a highly constrained Non-Maximum Suppression (NMS) intersection-over-union (IoU) threshold tailored specifically to tight cellular geometries. This configuration forces the YOLO detector to output highly isolated, membrane-hugging bounding box prompts. By systematically eliminating overlapping prompt configurations, we structurally prevent the downstream SAM decoder from merging adjacent clusters, effectively optimizing a cascaded pipeline for high-density instance separation. Consequently, this configuration serves a dual purpose: it stabilizes mask generation in dense environments and establishes a standardized, automated baseline to rigorously evaluate how standard geometric prompts translate to zero-shot segmentation boundaries when compared against specialized state-of-the-art frameworks.

##### YOLO

For detection, we employ YOLOv8n (where “n” denotes the lightweight “nano” variant) as the detection backbone to generate bounding box prompts. YOLOv8n was specifically selected to minimize the network’s parameter footprint (∼3.2 million parameters), ensuring that the overall pipeline remains highly efficient and executionally light when paired with the downstream Segment Anything Model (SAM) encoder. Input images are uniformly resized to a standard resolution of 256×256 pixels to match the network configuration. The network is trained for 300 epochs on the combined training split of the baseline datasets (comprising DSB18, Aureus, and T-cell cohorts) using a conservative learning rate of $1\times 10^{-5}$. This specific learning rate was empirically selected during validation to avoid catastrophic forgetting of the pretrained backbone features and to ensure stable convergence across high-density cell clusters. To refine predictions and reduce redundancy, we apply Non-Maximum Suppression (NMS), which filters overlapping bounding boxes and ensures that only the most confident detections are retained. These high-quality bounding boxes serve as reliable prompts for the subsequent segmentation step. The detection performance of YOLO across datasets is summarized in Table A1 (Appendix A), highlighting its ability to generalize across different imaging conditions.

##### SAM

For segmentation, we use the base version (ViT-B) backbone of the Segment Anything Model (SAM) as the mask predictor. The model is fine-tuned using the Dice Loss implementation from MONAI. Optimization is performed using the Adam optimizer applied exclusively to the mask decoder parameters, with a learning rate of $1\times 10^{-5}$ and no weight decay. The vision encoder, image encoder, and prompt encoder parameters are frozen during training to leverage pretrained representations while reducing computational cost. This configuration allows SAM to effectively leverage bounding box prompts and produce precise instance-level segmentations.

#### 3.3.2. Compared Methods

- StarDist [10]: A U-Net–based model that represents cells as star-convex polygons by regressing radial distances from object centers. StarDist was trained from scratch on our curated training dataset to establish its specialized, localized baseline. Candidate polygons are filtered with non-maximum suppression (NMS) to resolve overlaps, enabling accurate segmentation in crowded conditions with strong cell-to-cell contact. We chose StarDist as the reference task-specific model because it is highly effective at separating overlapping nuclei or cells, particularly in dense microscopy images, and has demonstrated robust performance across multiple datasets and imaging modalities. Its geometric representation is especially suitable for roughly convex cell shapes.
- Cellpose-SAM [12]: A hybrid approach that integrates SAM with Cellpose. SAM’s pretrained image encoder extracts robust general-purpose features from raw microscopy images, while the Cellpose decoder predicts vector flow fields, which are then converted to instance masks. This workflow allows dense segmentation of all cells in an image in a single pass, avoiding SAM’s sequential, prompt-based mask generation. The released base model was fine-tuned on diverse datasets, including the Cellpose dataset (covering fluorescence, brightfield, and phase-contrast modalities), TissueNet, and bacterial datasets such as DeepBacs, enhancing its generalization to different cell types and imaging conditions. Additionally, this network was retrained on our curated training dataset to establish its domain-specific benchmark.
- CellSAM [11]: A microscopy-focused adaptation of SAM that combines an object detector (CellFinder) with SAM for automatic prompt generation. CellFinder uses the Anchor-DETR61 framework with the same ViT backbone as the SAM module. This design improves sensitivity to faint boundaries and densely packed cells, enabling automatic, high-quality prompt generation. The CellSAM model was trained on multiple datasets, including the Cellpose cellular dataset, DSB18, TissueNet, Omnipose, DeepBacs, and MoNuSeg, supporting strong zero-shot generalization and rapid few-shot adaptation across diverse imaging modalities. While local constraints prevented retraining this specific framework, CellSAM was evaluated out-of-the-box for inference; the presence of DSB18 and DeepBacs in its original pre-training corpus ensures a highly equitable domain-level baseline comparison against our locally retrained architectures.

Despite the inclusion of these broader training domains (such as DeepBacs or TissueNet), we explicitly clarify that none of the target frames from our independent Breast and Flow Chamber evaluation sets was included in any model’s pre-training or fine-tuning pipelines, ensuring a strict zero-shot benchmark.

### 3.4. Evaluation Metrics

Model performance is evaluated using Average Precision (AP) across IoU thresholds from 0.50 to 0.90 in increments of 0.05. This metric captures the trade-off between precision and recall over varying detection confidence thresholds. AP is particularly suited for instance segmentation across diverse datasets, imaging modalities, and cell morphologies, where accurate localization and delineation of individual objects are crucial.

(7) $$\begin{array}{cc} AP(\tau ) & =\int_{0}^{1}p\left(r;\tau \right)\;dr \end{array}$$

### 3.5. Results

**Figure 5:**
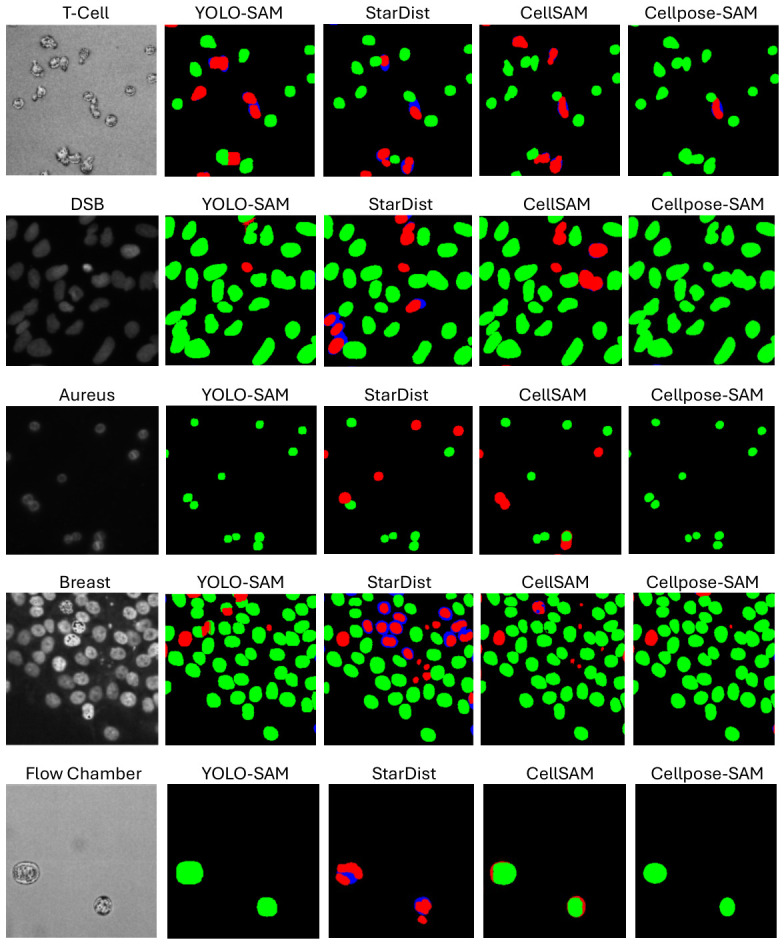
Predicted masks for a representative image of each dataset (threshold = 0.7). Green indicates True Positives (TP); red indicates False Positives (FP) and blue indicates False Negatives (FNs).

Table 5 summarizes the aggregated model performance by reporting the mean Average Precision (${\mathrm{mAP}}_{\left[0.50:0.90\right]}$) across the entire spectrum of evaluation datasets, while a comprehensive, threshold-by-threshold breakdown of individual AP values from IoU τ=0.50 to τ=0.90 is provided in Table A2 within Appendix A. Overall, Cellpose-SAM shows stronger performance in every dataset compared to the rest of the models. Figure 5 shows the predicted masks from all evaluated models across each of the five datasets.

On the T-cell dataset, all models perform competitively; however, Cellpose-SAM consistently achieves the highest AP across thresholds. Among the remaining models, the three of them perform very similar but YOLO-SAM maintains stronger performance than both StarDist and CellSAM as thresholds increase. This highlights the difficulty of accurately segmenting the shape of varying cells.

For the DSB18 dataset, performance is strong across all models. CellSAM and YOLO-SAM achieve high AP at lower thresholds, but Cellpose-SAM demonstrates the most stable and consistently high performance at all thresholds. This shows that SAM-based adaptations provide both accuracy and robustness on this type of data with many different cell shapes and sizes.

The Aureus dataset highlights challenges with small, densely packed bacteria. Here, Cellpose-SAM achieves the best and most consistent AP across thresholds again, while the rest show decent performance at lower thresholds but Stardist and CellSAM decline more sharply at stricter IoUs. This underlines the ability of Cellpose-SAM and YOLO-SAM to delineate cells more accurately.

On the Breast dataset, all SAM-based models achieve are very close at all thresholds outperforming StarDist by a clear margin. These results suggest that foundation models are better suited for dense fluorescence nuclei where accurate boundary delineation is essential.

Finally, the Flow Chamber dataset, designed to test robustness under dynamic conditions, exposes the largest performance gaps. While CellSAM achieves strong AP at lower thresholds, Cellpose-SAM delivers more stable performance at stricter IoUs, making it the most reliable model in this challenging setting. Again YOLO-SAM shows more stability along all thresholds but this time performs worse. StarDist struggles in this scenario, showing really poor generalization to brightfield sequences with motion and deformation.

Overall, SAM-based models consistently outperform StarDist across all datasets, particularly in cross-domain settings. StarDist struggles with irregular or non-rounded shapes due to its reliance on a star-convex polygon representation, whereas SAM-based methods capture object boundaries more faithfully and yield better shape representations. Among the SAM variants, YOLO-SAM and CellSAM achieve very similar performance, with CellSAM showing particular strength in lower thresholds but YOLO-SAM showing strength in higher ones.

This high-threshold resilience in YOLO-SAM is deeply tied to the localized precision of its bounding box prompts. As demonstrated by our detection-to-segmentation correlation analysis (Table A1), when the YOLO backbone successfully locks onto a target, the performance gap (Δ) between the prompt and the final strict ${\mathrm{AP}}_{75}$ mask remains remarkably tight—averaging a drop of only −0.074 on unseen fluorescence data (Breast dataset) and −0.162 on challenging brightfield data (Flow Chamber). This tight translation indicates that YOLO-SAM’s ultimate performance bottleneck is dictated by initial object detection precision rather than a breakdown in SAM’s mask generation capacity.

**Figure 6:**
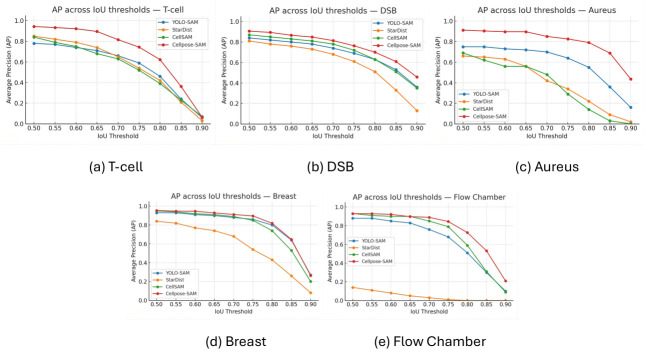
AP performance across IoU thresholds (τ) for different models on all datasets. (**a**) T-cell dataset, (**b**) DSB dataset, (**c**) Aureus dataset, (**d**) Breast dataset, (**e**) Flow Chamber dataset.

However, Cellpose-SAM consistently emerges as the best and most robust model, maintaining stability across thresholds and object densities (see Figure 6). Crucially, this robust accuracy does not come at the expense of operational efficiency; as detailed in the computational benchmarks in Table A3, Cellpose-SAM maintains an optimized inference speed (598.87 ms) that outperforms generalist alternatives like CellSAM and the multi-stage YOLO-SAM pipeline, both of which introduce a heavy execution latency penalty over 2.7 s. These findings highlight the value of adapting foundation models like SAM for microscopy, while also emphasizing the importance of domain-aware priors to maximize performance across diverse imaging conditions.

### 3.6. Discussion

In this study, we systematically evaluated segmentation models across microscopy datasets that span both brightfield and fluorescence modalities, as well as diverse cell populations ranging from bacteria to mammalian cells. This heterogeneity allowed us to test models under varying conditions of contrast, shape complexity, and object density—factors that closely mirror real-world biomedical imaging challenges.

Across these settings, SAM-based methods consistently outperformed StarDist, especially in cross-domain applications where training and testing conditions differed. This performance gap reflects the inherent limitations of StarDist’s star-convex polygon representation, which struggles with irregular or elongated morphologies, compared to the more flexible mask proposals generated by SAM.

Among the SAM variants, YOLO-SAM and CellSAM achieved similar overall performance, though with subtle differences depending on thresholding criteria and imaging modality. Cellpose-SAM, however, stood out as the most stable and reliable approach, preserving performance across datasets, thresholds, and varying cell densities. This suggests that coupling SAM’s generalist capabilities with Cellpose’s domain-specific priors is particularly effective for microscopy segmentation.

Future progress will likely depend on three fronts: (i) enhanced domain adaptation strategies, including fine-tuning or self-supervised pretraining on modality-specific data; this is particularly crucial for extending the framework’s cross-domain generalization to highly distinct and challenging structural modalities such as histopathology, phase-contrast, and electron microscopy, as well as mitigating variations introduced by unseen laboratory staining protocols and imaging artifacts; (ii) improving annotation efficiency through weak supervision or interactive pipelines; (iii) incorporating biological context such as size distributions, shape constraints, or temporal consistency in dynamic experiments, so that models leverage not only pixel-level information but also biologically meaningful context to improve robustness and interpretability.

In summary, this study bridges the gap between traditional architectures and emerging foundation models by validating the paradigm shift toward universal microscopy segmentation. Our systematic benchmarking confirms that while traditional frameworks like StarDist offer lightweight deployment, they remain structurally constrained by geometric assumptions. Conversely, foundation model derivatives seamlessly absorb cross-domain variations in contrast, modality, and morphology without requiring localized re-training. By mapping the distinct operational trade-offs of frameworks like YOLO-SAM, CellSAM, and the highly stable Cellpose-SAM, these interconnected findings translate empirical data into an actionable deployment blueprint—demarcating the current limits of zero-shot generalization while charting a clear path toward domain-aware foundation pipelines in high-throughput biomedical workflows.

Overall, our findings indicate that foundation model–based approaches already offer a strong step toward universal segmentation in microscopy, but their full potential will be realized only when paired with domain-aware refinements and evaluated systematically across heterogeneous imaging scenarios.

### 3.7. Limitations

While this evaluation highlights the strong potential of foundation models, several distinct limitations should be factored into the interpretation of our comparative trends:

- Dataset Scale and Sample Size Constraints: The validation sets utilized are relatively small in scale. While cohorts like the Aureus set (5 images) and the Breast cancer dataset (16 images) serve as valuable benchmarks for preliminary zero-shot cross-domain exploration, small sample sizes are inherently susceptible to pronounced statistical fluctuations. Local anomalies—such as an image with unusually dense cellular clustering, severe regional focus artifacts, or atypical staining intensities—can disproportionately skew the aggregate mean Average Precision (mAP) or runtime metrics. Consequently, these findings should be treated as a localized proof-of-concept demonstrating architectural potential, rather than an absolute guarantee of large-scale mathematical stability across massive, high-throughput imaging pipelines.
- Absence of Statistical Significance Metrics: A related methodological restriction concerns the lack of statistical significance testing or error bars for the reported Average Precision (AP) and mAP metrics. Because the benchmarked foundation models leverage frozen, pre-trained weights evaluated in a zero-shot inference paradigm, their outputs are entirely deterministic for a given input. Consequently, the models were evaluated over a single operational run. In the absence of stochastic resampling methods (such as bootstrapping or multi-seed cross-validation), localized metric discrepancies between models should be interpreted as localized observations within this cohort rather than definitive statistical superiorities.
- Boundary Sensitivity in Complex Regions: Despite their overall strengths, all SAM-based methods exhibited a sensitivity to fine boundary details in densely packed or low-contrast regions. This points to a broader, systemic trade-off between the extensive generalization capacity of large-scale foundation models and the localized pixel-level precision traditionally afforded by domain-specialized approaches.
- Dimensional and Temporal Restrictions: The current evaluation of the proposed framework is strictly restricted to 2D static micrographs. In fields such as 3D volumetric segmentation or temporal cell tracking, volumetric reconstruction and frame-to-frame association rely heavily on the accuracy of slice-by-slice or static frame segmentations. While the robust instance separation achieved here lays a critical foundation for multi-dimensional workflows, extending this automated pipeline to natively handle z-stacks and time-lapse data represents a key future direction required to fully assess model generalizability across spatial and temporal dimensions.

## 4. Summary and Conclusions

This review has surveyed the rapidly evolving field of microscopy cell segmentation, tracing its trajectory from classical image-processing pipelines to specialized deep learning models and, more recently, to the emergence of foundation models. Advances in deep learning, particularly architectures such as U-Net, Mask R-CNN, and their derivatives, have enabled major progress in tackling the challenges posed by diverse cell morphologies and imaging conditions. At the same time, the availability of public datasets and competitions—such as the Data Science Bowl (DSB) and the NeurIPS Cell Segmentation Challenge—has played a pivotal role in driving innovation, providing standardized benchmarks, and fostering reproducibility. While many approaches have been proposed across different modalities, we focused in particular on brightfield and fluorescence microscopy, which remain the most widely used and challenging in practice.

Given the diversity of approaches and the variation in metrics and datasets used across studies, a fair and standardized comparison of models is essential. To this end, we conducted a controlled experimental evaluation using brightfield and fluorescence images from multiple cell types. Four representative approaches were benchmarked: StarDist, a geometry-aware deep learning model widely used in microscopy; CellSAM, a SAM adaptation to cellular imaging; Cellpose-SAM, a hybrid that couples the generalization of SAM with Cellpose’s robustness to diverse morphologies; and YOLO-SAM, our proposed detection-driven prompting strategy that integrates YOLO for the cell detection with SAM for improved instance segmentation.

Overall, foundation models represent a promising step toward universal, cross-domain cell segmentation, with the potential to reduce manual labeling effort and adapt across diverse modalities. Future progress demands models that generalize robustly across both cell types and imaging techniques, supported by large, high-quality datasets and benchmarks that better capture real-world variability. Achieving these goals will be key for building practical, automated workflows that are accurate, reproducible, and trustworthy in biomedical applications.

By combining a comprehensive review with experimental benchmarking, this work provides both conceptual and practical perspectives. It underscores the opportunities and challenges in advancing microscopy cell segmentation and offers guidance for researchers seeking to develop or adopt effective strategies in this rapidly progressing field.

## Figures and Tables

**Table 1:** Overview of Instance Segmentation Models in Biomedical Imaging.

Model	Year	Domain	Key Features	Reference
Mask R-CNN	2017	General/Biomedical	Region proposals + mask head	[8]
SEGAN	2017	Biomedical	ConvLSTM + adversarial training	[90]
StarDist	2018	Microscopy	Star-convex polygon representation of instances	[10]
HoVer-Net	2019	Histology	Horizontal/vertical distance maps for nuclear separation	[91]
Cellpose	2021	Microscopy	Vector flow fields; modality-agnostic	[23]
MESMER	2022	Histology	Generalist for multiplexed tissue images	[24]
Cellpose 2.0	2022	Microscopy	User training, style transfer	[92]
LACSS	2022	Microscopy	Weak supervision for instance masks	[93]
Omnipose	2022	Microscopy	Flexible contours; excels on bacteria/irregular shapes	[83]
BriFiSeg	2023	Microscopy	Multi-scale features for gland segmentation	[94]
CPP-Net	2023	Microscopy	Contour proposal network for dense scenes	[95]
Cellulus	2023	Microscopy	Self-supervised + multi-scale features for heterogeneity	[96]
Cellpose 3.0	2025	Microscopy	3D support, improved training UI	[97]

**Table 2:** Foundation Models Adapted to Biomedical Imaging.

Model	Year	Domain	Key Features	Reference
Segment Anything (SAM)	2023	Generalist	Promptable segmentation; billion-mask pretraining	[9]
Cell-SAM	2023	Microscopy	SAM with domain-specific tuning for cells	[11]
MicroSAM	2023	Microscopy	SAM adaptation for micro-scale, faint boundaries	[100]
Vista 2D	2025	Microscopy	SAM + contrastive learning for robust 2D microscopy	[101]
Cellpose-SAM	2025	Microscopy	Hybrid SAM + Cellpose pipeline	[12]

**Table 3:** Summary of Microscopy Datasets for Segmentation.

Name	Technique
DSB18 (Kaggle 2018) [27]	Fluorescence (nuclei)
Cellpose [23]	Fluorescence, Brightfield, Phase-contrast
LIVECell [107]	Phase-contrast (live cells)
TissueNet [24]	Immunofluorescence (multi-tissue, whole-cell + nuclei)
NeurIPS 2022 Challenge [29]	Mixed modalities
DeepBacs [108]	Bacterial (synthetic, BF, PC)
EVICAN [109]	Brightfield
ISBI Cell Tracking [6]	Time-lapse (2D + 3D)

**Table 4:** Datasets used for training and testing.

Dataset	Modality	Cell Type	# Images	Use
DSB18 [27]	Fluorescence	Various nuclei	447/50	Train/Test
Aureus [110]	DIC + fluorescence	*S. aureus*	28/5	Train/Test
T-cell [111]	Brightfield	Mouse CD4+ T cells	174/35	Train/Test
Breast [112]	Fluorescence	DCIS.COM Lifeact-RFP	16	Test only
Flow chamber [113]	Brightfield	Pancreatic cancer cells	57	Test only

**Table 5:** Summary of mean Average Precision (${\mathrm{mAP}}_{\left[0.50:0.90\right]}$) across models and datasets.

Model	T-Cell	DSB	Aureus	Breast	Flow Chamber	Overall Average
YOLO-SAM	0.557	0.688	0.596	0.791	0.643	0.655
StarDist	0.561	0.593	0.399	0.573	0.047	0.435
CellSAM	0.544	0.706	0.374	0.770	0.697	0.618
Cellpose-SAM	0.699	0.762	0.801	0.813	0.766	0.768

## Data Availability

The data presented in this study are available in Data Science Bowl (2018) at https://www.nature.com/articles/s41592-019-0612-7 (accessed on 18 May 2025) https://zenodo.org/records/5550933 (accessed on 19 May 2025), T cell dataset at https://zenodo.org/records/4034929 (accessed on 19 May 2025), Breast cancer cell dataset at https://zenodo.org/records/4034976 (accessed on 29 May 2025) and Flow chamber dataset at https://zenodo.org/records/4034939 (accessed on 29 May 2025). The code used for this study is available on our GitHub repository at https://github.com/diegomartiperezz/MicroscopyCellSegmentation.git (accessed on 27 June 2026).

## Appendix A. Additional Tables

Table A1 summarizes the detection performance of the YOLOv8 model across different microscopy datasets, which we correlate directly with downstream segmentation success to isolate pipeline bottlenecks. The results demonstrate that YOLO achieves consistently high precision and recall, indicating reliable generalization to diverse imaging modalities.

Crucially, cross-referencing these detection metrics with our ultimate segmentation accuracy reveals distinct modality behaviors. For the out-of-distribution fluorescence cohort (Breast dataset), we observe a near-perfect translation from detection to mask generation, yielding a minimal performance gap (Δ=−0.074) at a strict ${\mathrm{AP}}_{75}$ threshold. Conversely, on the dynamic brightfield cohort (Flow Chamber), the analysis proves that the marginal performance drop is primarily a cascading limitation of initial detection precision (0.827) under low-contrast halo conditions, rather than a failure of the SAM mask decoder itself. Once a high-quality bounding box is generated (supported by a high detection recall of 0.891), the downstream foundation model successfully resolves the challenging brightfield boundaries with a tight performance gap (Δ=−0.162) that outperforms baseline fluorescence sets like the T-cell cohort (Δ=−0.249).

For a highly detailed, granularity-focused breakdown of model performance across varying strictness levels, Table A2 provides the complete set of individual Average Precision (AP) scores. This comprehensive data matrix spans Intersection over Union (IoU) thresholds from τ=0.50 to τ=0.90 in steps of 0.05 across all five evaluated microscopy datasets, serving as the raw baseline data from which the aggregated mean Average Precision (mAP) summary trends in the main text are derived.

**Table A1:** Correlation: YOLO Detection vs. SAM Segmentation (${\mathrm{AP}}_{75}$).

Dataset	Det. Prec.	Det. Rec.	Det. $F_{1}$	Seg. ${\mathrm{AP}}_{75}$	Gap (Δ)
T-cell	0.890	0.795	0.839	0.590	−0.249
DSB	0.941	0.846	0.886	0.690	−0.196
Aureus	0.956	0.750	0.824	0.640	−0.184
Breast	0.944	0.926	0.934	0.860	−0.074
Flow Chamber	0.827	0.891	0.842	0.680	−0.162

**Table A2:** Average Precision (AP) across different thresholds.

	Threshold τ								
**Model**	**0.50**	**0.55**	**0.60**	**0.65**	**0.70**	**0.75**	**0.80**	**0.85**	**0.90**
T-Cell									
YOLO-SAM	0.78	0.77	0.74	0.71	0.66	0.59	0.46	0.24	0.06
StarDist	0.85	0.82	0.79	0.74	0.65	0.54	0.42	0.21	0.03
CellSAM	0.84	0.79	0.75	0.68	0.63	0.52	0.39	0.23	**0.07**
Cellpose-SAM	0.94	0.93	0.92	0.90	0.82	0.74	0.62	0.36	0.06
DSB									
YOLO-SAM	0.84	0.82	0.80	0.78	0.74	0.69	0.63	0.53	0.36
StarDist	0.81	0.78	0.76	0.73	0.68	0.61	0.51	0.33	0.13
CellSAM	0.87	0.85	0.83	0.81	0.78	0.72	0.63	0.51	0.35
Cellpose-SAM	0.91	0.89	0.87	0.85	0.81	0.76	0.70	0.61	0.46
Aureus									
YOLO-SAM	0.75	0.75	0.73	0.72	0.70	0.64	0.55	0.36	0.16
StarDist	0.66	0.65	0.63	0.56	0.42	0.34	0.22	0.09	0.02
CellSAM	0.69	0.62	0.56	0.56	0.48	0.29	0.14	0.03	0.00
Cellpose-SAM	0.91	0.90	0.90	0.90	0.85	0.83	0.79	0.69	0.44
Breast									
YOLO-SAM	0.93	0.93	0.91	0.90	0.88	0.86	0.80	0.64	**0.27**
StarDist	0.84	0.82	0.77	0.74	0.68	0.54	0.43	0.26	0.08
CellSAM	0.95	0.94	0.92	0.91	0.89	0.85	0.74	0.53	0.20
Cellpose-SAM	0.95	0.95	0.95	0.93	0.91	0.90	0.82	0.65	0.26
Flow_Chamber									
YOLO-SAM	0.88	0.88	0.85	0.83	0.76	0.68	0.51	0.30	0.10
StarDist	0.14	0.11	0.08	0.05	0.03	0.01	0.00	0.00	0.00
CellSAM	0.93	0.91	0.90	0.90	0.85	0.79	0.59	0.31	0.09
Cellpose-SAM	0.93	0.93	0.92	0.90	0.89	0.85	0.73	0.53	0.21

To evaluate the operational requirements of the benchmarked frameworks, we measured their computational footprint during inference on the Aureus dataset. As summarized in Table A3, the architectural complexity of the models introduces significant trade-offs between segmentation performance, processing speed, and memory consumption. StarDist operates as the most lightweight baseline, requiring only 152.77 ms per image with a peak memory allocation of 1290.67 MB. Among the foundation-scale models, Cellpose demonstrates remarkable optimization, processing images in 598.87 ms while maintaining a moderate memory profile (2110.58 MB). Conversely, the generalist SAM-based models demand substantially higher computational resources; CellSAM requires 2717.47 ms per image, while the cascaded YOLO-SAM pipeline exhibits the longest processing latency at 5497.67 ms per image, though both settle at a comparable peak memory ceiling of approximately 3215 MB. This indicates that while foundation models deliver high cross-domain robustness, their deployment carries a significant latency penalty that must be factored into high-throughput production environments.

**Table A3:** Computational Efficiency Benchmark on the Aureus Dataset.

Model	Mean Inference Time (ms/Image)	Max Peak Memory (MB)
YOLO-SAM	5497.67	3214.56
StarDist	152.77	1290.67
CellSAM	2717.47	3217.91
Cellpose	598.87	2110.58
